# The breathless swimmer: could this be swimming-induced pulmonary edema?

**DOI:** 10.1186/s40798-018-0166-8

**Published:** 2018-11-19

**Authors:** James H. Hull, Mathew G. Wilson

**Affiliations:** 1 Department of Respiratory Medicine, Royal Brompton Hospital, London, SW3 6HP UK; 2 Athlete Health and Performance Research Centre, Aspetar Orthopaedic and Sports Medicine Hospital, Doha, Qatar

**Keywords:** Athlete, Cough, Dyspnea, Swimming, Heart

## Abstract

Swimming-induced pulmonary edema is an infrequently encountered cause of acute respiratory distress in open-water swimmers. The condition can be challenging, with athletes often wanting answers to three main questions regarding (i) a definitive and robust diagnosis, (ii) the risk of recurrence, and (iii) what can be done to avoid recurrence. This commentary provides an overview of the best available evidence, in light of a recently published systematic review.

## Key points

- Swimming induced pulmonary edema (SIPE) is a rare but important cause of acute respiratory distress in swimmers.
- SIPE can be difficult to diagnose and athletes with this condition often report non-specific clinical features that can resolve over a highly variable time-course.
- There is currently considerable uncertainty regarding the best assessment and treatment algorithms for suspected SIPE.
- This commentary reviews the key characteristics and features of SIPE, as addressed in a recent systematic review of the published data, in this area.

## Commentary

Assessing the acutely breathless athlete is challenging. The differential diagnosis is broad and confounded by the non-specific nature of symptoms such as cough and wheeze, and poor predictive clinical signs. Recognised causes of acute breathlessness in athletes not only include prevalent respiratory conditions, such as exercise-induced asthma (EIA) and exercise-induced laryngeal obstruction (EILO), but also cardiac disease and transient conditions that impair cardiorespiratory function [1].

Swimming-induced pulmonary edema (SIPE) is a type of immersion pulmonary edema that occurs when fluid accumulates in the lungs during surface or underwater swimming [2]. Whilst SIPE routinely appears in the standard list of differential diagnoses for the acutely breathless athlete, most clinicians will have never seen this condition. The literature in this area is disjointed, with data from scuba and immersion divers and recreational triathletes seemingly admixed. Consequently, there are no go-to guidelines or established consensus recommendations on who is best placed to assess and manage a potential case of SIPE. Indeed, when SIPE is raised as a potential diagnosis, it is highly likely that the attending clinician has never seen a case and has limited or no idea what to do next. Yet for the athlete, the development of potential SIPE has major and immediate implications, not only raising anxiety regarding their cardiopulmonary health but also raising concern regarding continuation of their athletic career (Table 1) [3]. Quite rightly, clinicians should be prepared to answer three real and pressing questions: (i) was it definitely SIPE? (ii) will it recur? and (iii) what can be done to avoid/treat it?

**Table 1:** Key considerations for the clinician addressing an athlete with probable swimming-induced pulmonary edema (SIPE)

Key questions	Evidence/comment	Action/recommendations
Was it SIPE?	• Clinical features are non-specific and a variety of features (e.g. cough, crackles, desaturation) are present in two thirds cases. • Symptoms occur in close temporal association with water immersion. • Rapid resolution (usually complete < 48 h) appears to be a universal feature and may be of use in differentiating SIPE from aspiration. • Chest radiograph/ultrasound evidence of pulmonary edema is informative.	• Obtain a full and thorough collateral history, including assessment of timing of onset of symptoms. • Evaluate any chest imaging and blood results. • Utilise logical investigations to rule out other differential diagnoses (e.g. bronchoprovocation testing, continuous laryngoscopy during exercise [18]).
Will it recur?	• Recurrence rates are variable (between 13 and 40%) but the condition can definitely recur.	• Counsel the athlete that there is a reasonable chance of recurrence and provide safety advice for both athlete and medical teams. • Stepwise return to swimming.
What can be done to avoid/treat it?	• Several risk factors are recognised. • Detection of underlying cardiorespiratory disease is important	• Rule out underlying cardiac or respiratory disease process, e.g. by arranging echocardiogram, blood pressure monitor, lung function + more detailed investigations as indicated. • Avoid over-hydration, tight fitting wetsuits, fish oil and aspirin. • Consider the use of oral sildenafil prior to exercise in selected and well-characterised cases. This medication is not prohibited currently.

We therefore welcome Spencer and colleagues’ [4] timely, thorough, clinically informative and well-conducted systematic review of the literature on this topic. The authors conducted an extensive literature review, identifying and summarising the albeit relatively limited evidence base (*n* = 9 studies after application of exclusion criteria). Their overall aim was to address a number of key clinical questions pertaining to SIPE, with a synthesis of the data regarding epidemiology, risk factors, outcome(s) and effectiveness of treatment. Whilst any outcome analysis is only as good as the quality of the best data available, their review not only helpfully consolidates the current state of knowledge but also, most importantly, acts to identify current deficits.

While SIPE appears to be a relatively uncommon condition, it arguably has significant public health implications All epidemiological evaluations are confounded by the case variable definition applied, but there are a concerning number of studies that appear to define SIPE simply based on an athlete’s ‘self-report’ of cough and breathlessness development whilst swimming. Nevertheless, the best available data would appear to indicate that SIPE affects 1–2% of all triathletes [5]. Smith at al. [6] reported a lower incidence rate of < 1% based upon medical records from ~ 70,000 UK-based triathlon competitions. Consequently, the overall incidence and prevalence rates of SIPE reported to date appear to indicate that it is a relatively uncommon condition, especially when considered in the context of the other main differential diagnoses, such as EIA (up to 70% in some series [7]) and EILO (approx. 5–9% [8]). However, SIPE prevalence rates are still in excess of other serious health conditions affecting athletes, such as inherited cardiac pathology, and if not identified or appropriately managed, the condition is of serious concern.

At present, risk factors for the development of SIPE are difficult to ascertain, as methodological concerns and study heterogeneity limit data synthesis in this context [4]. An association between hypertension and SIPE has long been highlighted [2, 9] on the basis that the development of potential diastolic dysfunction may alter cardiac filling, resulting in altered pulmonary vascular pressures and promoting extravasation. Moon and colleagues [10] reported a heightened prevalence of left ventricular hypertrophy in open-water swimmers who succumbed to possible SIPE, but these findings may simply be in keeping with a longer-term response to underlying cardiovascular stress. Other SIPE risk factors worthy of further exploration include the use of medications or supplements that could potentially impair platelet function and vasodilatory function, such as fish oil or aspirin [4].

Perhaps the most pertinent and important question facing clinicians is how best to diagnose SIPE. In keeping with the other differential diagnoses such as respiratory disorders, clinical diagnostic accuracy is confounded by the poor predictive value of what are essentially non-specific symptoms [11]. Indeed, it appears that generally the rule of two thirds seems to apply; specifically, haemoptysis or at least blood tinged sputum is present in approximately two thirds of SIPE cases, pulmonary auscultation reveals crackles in two thirds, oxygen saturation is reduced in two thirds and a chest radiograph is abnormal in two thirds of cases [12]. A more consistent and almost universal feature is the complete resolution of symptoms in under 48 h. More recent reports highlight the value of point-of-care testing, including ultrasound to identify pulmonary interstitial fluid accumulation [13]. The role of other diagnostic tests or biomarkers is yet to be established, but there is evidence that obvious go-to biomarkers of cardiac dysfunction are not elevated in individuals presenting with SIPE [14]. Regardless, it is clear that a currently unmet need is a diagnostic questionnaire or screening tool for SIPE.

Acute treatment reported as administered in treated cases includes the immediate removal from water, oxygen supplementation, inhaled beta-2 agonists and judicious use of diuretics. Recent pathophysiology studies have suggested a stroke volume mismatch between the right and left ventricles [15] and a raised mean pulmonary arterial pressure and pulmonary arterial wedge pressure [16]. Consequently, the latter can be modulated by the administration of sildenafil, a selective inhibitor of phosphodiesterase-5, that results in relaxation of vascular smooth muscle, acting to reduce pulmonary vascular resistance during exercise. Finally, a case report in a confirmed case of SIPE cites the efficacy of a single dose of 50 mg oral sildenafil taken prior to a triathlon for mitigating the recurrence [17].

So, where does this leave the clinician facing a potential case of SIPE? Clearly, any discussion with an athlete regarding his or her health should be fully informed and based on the current best state of knowledge (or lack of in certain aspects). Moreover, given the current knowledge deficits, this discussion should be candid regarding the limitations of a clinical-based approach to diagnosis, risk of recurrence, and treatment options. Recent advances in understanding of the pathophysiology of SIPE offer promise in terms of future treatment to prevent re-occurrence.

## Abbreviations

EIA: Exercise-induced asthma
EILO: Exercise-induced laryngeal obstruction
SIPE: Swimming-induced pulmonary edema
